# Modeling the Effects of Varying the Ti Concentration on the Mechanical Properties of Cu–Ti Alloys

**DOI:** 10.1021/acsomega.3c07561

**Published:** 2024-02-19

**Authors:** Vasileios Fotopoulos, Corey S. O’Hern, Mark D. Shattuck, Alexander L. Shluger

**Affiliations:** † Department of Physics and Astronomy, University College London, Gower Street, London WC1E 6BT, U.K.; ‡ Department of Mechanical Engineering & Materials Science, Yale University, New Haven, Connecticut 06520, United States; § Department of Physics, Yale University, New Haven, Connecticut 06520, United States; ∥ Department of Applied Physics, Yale University, New Haven, Connecticut 06520, United States; ⊥ Benjamin Levich Institute and Physics Department, The City College of the City University of New York, New York, New York 10031, United States; # WPI-Advanced Institute for Materials Research (WPI-AIMR), Tohoku University, 2-1-1 Katahira, Aoba-ku, Sendai 980-8577, Japan

## Abstract

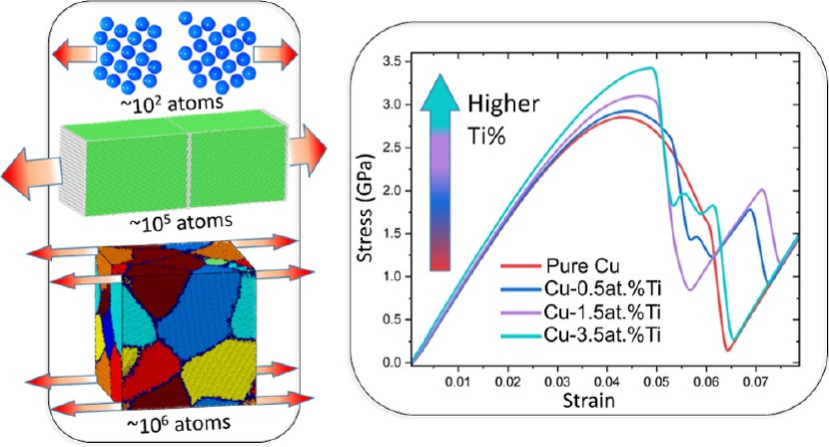

The mechanical properties of CuTi alloys have been characterized extensively through experimental studies. However, a detailed understanding of why the strength of Cu increases after a small fraction of Ti atoms are added to the alloy is still missing. In this work, we address this question using density functional theory (DFT) and molecular dynamics (MD) simulations with the modified embedded atom method (MEAM) interatomic potentials. First, we performed calculations of the uniaxial tension deformations of small bicrystalline Cu cells using DFT static simulations. We then carried out uniaxial tension deformations on much larger bicrystalline and polycrystalline Cu cells by using MEAM MD simulations. In bicrystalline Cu, the inclusion of Ti increases the grain boundary separation energy and the maximum tensile stress. The DFT calculations demonstrate that the increase in the tensile stress can be attributed to an increase in the local charge density arising from Ti. MEAM simulations in larger bicrystalline systems have shown that increasing the Ti concentration decreases the density of the stacking faults. This observation is enhanced in polycrystalline Cu, where the addition of Ti atoms, even at concentrations as low as 1.5 atomic (at.) %, increases the yield strength and elastic modulus of the material compared to pure Cu. Under uniaxial tensile loading, the addition of small amounts of Ti hinders the formation of partial Shockley dislocations in the grain boundaries of Cu, leading to a reduced level of local deformation. These results shed light on the role of Ti in determining the mechanical properties of polycrystalline Cu and enable the engineering of grain boundaries and the inclusion of Ti to improve degradation resistance.

## Introduction

Numerous experimental studies have shown that binary alloys made from Cu have significantly improved mechanical properties compared to pure Cu.^1,2^ For instance, Cu–Be alloys have been widely used in numerous applications.^3,4^ However, Be is highly toxic, even in small amounts, and thus, there is significant interest in identifying other Cu alloys with similar advantageous properties. In particular, Cu–Ti alloys, used in a wide range of applications,^5^ are promising since they possess high strength and electrical conductivity, as well as improved corrosion resistance.^6−8^ Furthermore, Cu–Ti alloys have an increased mechanical strength over pure Cu even at concentrations less than 5 at. % Ti.^9,10^

A detailed understanding of the atomistic mechanisms that improve the strength of Cu through the addition of small amounts of Ti can be achieved by using theoretical and computational modeling. Kohn–Sham^11,12^ density functional theory (DFT) and other quantum mechanics-based calculation methods that provide highly accurate electronic structure measurements for alloys.^13,14^ Several theoretical studies highlight the strengthening effect of metallic solutes when they are introduced into the grain boundaries (GBs) of metals, such as Cu,^15^ Ni,^16^ V,^17^ and Au.^18^ These calculations are limited to systems with only ∼100 atoms due to their computational cost.^19^ However, understanding the structural and mechanical properties of polycrystalline metals requires much larger system sizes than ∼100 atoms.^20^ Large-scale molecular dynamics (MD) simulations using embedded atom method (EAM) potentials can reproduce the structural and mechanical properties of many alloys.^21^ The modified EAM [modified embedded atom method (MEAM)] proposed by Baskes^22^ was developed to extend EAM interatomic potentials to alloys with strong angular bonding. MD simulations using MEAM potentials have been carried out to understand important properties of alloys, such as ductile versus brittle mechanical response,^23^ structure–property relationships,^24^ dislocation dynamics,^25^ and fracture mechanics,^26^ in FCC metals and alloys. However, MEAM potentials have been developed for only a small fraction of alloys.^27,28^

Several previous studies have investigated the effect of metallic dopants on the mechanical properties of Cu and similar metals using either DFT^29^ or semi-empirical MD simulations. However, identifying the origins of the experimentally observed improvements in the mechanical properties of polycrystalline Cu–Ti compared to those of pure Cu requires an understanding of the role of topological defects, such as dislocations. In this study, we investigate how the addition of Ti atoms affects the mechanical properties of bicrystalline and polycrystalline Cu using both DFT calculations and MEAM MD simulations. First, we use DFT calculations involving relatively small periodic cells to assess the accuracy of a recent MEAM potential that was fitted for Cu–Ti.^30^ We find that the MEAM potential accurately predicts the most energetically favorable segregation sites of Ti at the Cu GBs. Using DFT calculations of CuTi alloys undergoing uniaxial tension deformations, we show that the addition of Ti increases charge localization and the separation energy of Cu GBs, which indicates the formation of covalent bonding between Ti and its neighboring Cu atoms. We then carried out MEAM MD simulations of bicrystalline Cu systems with Ti impurities undergoing uniaxial tension deformation. We show that, similar to the DFT results, the presence of Ti increases the yield strength of bicrystalline Cu by hindering the formation of stacking faults. We then conduct MEAM MD simulations of polycrystalline Cu–Ti undergoing uniaxial tension deformations. We find that the addition of even a small amount of Ti increases the yield strength and Young’s modulus of the Cu polycrystals. The increases in the yield strength and elastic modulus of polycrystalline Cu are caused by the fact that Ti hinders the emission of Shockley dislocations from the GBs during the tensile deformation. This strengthening increases with the concentration of Ti atoms in the GBs. In light of recent experimental studies that developed advanced materials with controllable interfaces,^31^ our findings highlight the potential of decorating GBs in polycrystalline Cu with Ti atoms to improve its resistance to degradation.

## Methods

### First-Principles Calculations

**Figure 1:**
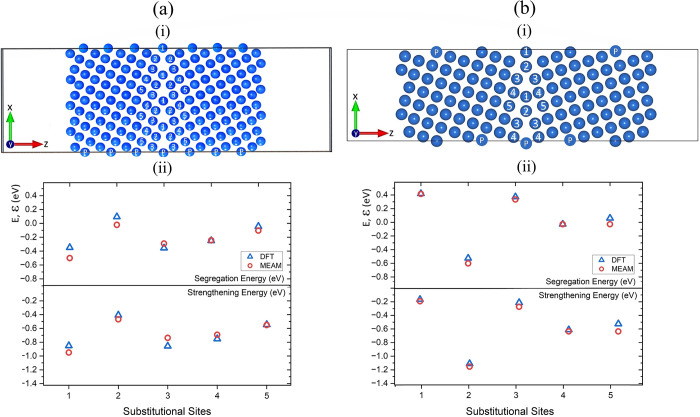
(a) (i) Cu 296-atom (210)[100] Σ5 GB simulation cell. Cu atoms are shown in blue, and the numbered atoms correspond to the substitutional Ti sites. Atoms labeled with a ‘P’ represent atoms that are periodically replicated. (ii) Comparison of Ti segregation (top) and strengthening energies (bottom) obtained using DFT and MEAM for the five substitutional sites. (b) (i) Cu 232-atom (310)[001] GB simulation cell for the labeled segregation sites. (ii) Comparison of Ti segregation (top) and strengthening energies (bottom) from DFT and MEAM.

The DFT calculations were carried out using the Vienna Ab Initio Simulation Package^32−34^ with the Perdew–Burke–Ernzerhof (PBE) GGA exchange-correlation functional^35^ in 76- and 108-atom periodic cells (additional details concerning the DFT calculations can be found in Appendix A). In line with previous studies of Cu,^36^ a mixture of the Davidson^37^ and RMM-DIIS^38,39^ algorithms was used to minimize the energy. Relevant details about the functional form of the ground state energy () obtained via DFT/PBE can be found in the literature.^35^ Two Σ5 twin boundary symmetries were examined, (210)[100] and (310)[001], using different cell sizes. These GBs possess among the lowest energies reported in Cu.^40^ The GB simulation cells were periodically replicated in the *x*-, *y*-, and *z*-directions. In the *z*-direction, 10 Å of vacuum is added to the simulation cell to avoid interactions between periodic images [see Figure 1a(i)]. For the 76-atom GB and 108-atom bulk cells, in line with previous studies,^41−43^ converged 5 × 4 × 1 and 4 × 4 × 4 *k*-point grids were used, respectively, with a 450 eV energy cutoff and a net force convergence criterion of 0.01 eV/Å, which is consistent with previous DFT calculations in Cu GBs.^29,44^ To determine the number of *k*-points for various cell sizes, the product of the length of the cell and the number of *k*-points in the *x*-, *y*-, and *z*-directions was chosen to be as close as possible to 35 *k*-points × Å to ensure a converged *k*-point sampling for all unit cells of different dimensions, given the Cu lattice constant of 3.62 Å. In the case of the GB cell, due to the added vacuum, just one *k*-point is used in the *z*-direction. The atomic positions were relaxed using energy minimization to an energy tolerance of <10^–5^ eV.

### Molecular Dynamics Simulations of Cu–Ti

All MD simulations were performed using the large-scale atomic molecular massively parallel simulator.^45^ 120,000-atom bicrystalline simulation cells of (210)[100] Σ5 GBs were constructed using the Atomsk code.^46^ For polycrystalline systems, we considered simulation cells with volumes of 8 × 10^6^ Å^3^ containing randomly oriented grains with 665,500 atoms. The polycrystals were then built using radical Voronoi tessellation of randomly distributed points or nodes.^46^ Radical Voronoi tessellation allows us to construct complex grain structures, such as curved GBs commonly found in experimental images of polycrystalline metals.^47,48^ Typically, 12 nodes were introduced randomly within the periodic cells. The grain sizes obtained from Voronoi tessellation follow a Gaussian distribution with a mean grain volume of 6.6 × 10^5^ Å^3^ (with a standard deviation of 0.1 × 10^6^ Å^3^) and a mean grain size (MGS) of approximately 89 Å. The MGS values were computed using the mean linear intercept method.^49^ This value for the MGS is in line with the grain sizes of experimentally reported nanocrystalline Cu^50,51^ and Cu-based alloys.^2^

Table 1 summarizes the MGS, temperature (T), strain rate, and Young’s modulus used in the MD simulations, together with the values from previous theoretical and experimental studies of polycrystalline Cu. Table 1 illustrates that the elastic moduli obtained from the EAM and MEAM MD simulations were close to the respective values reported in the experimental studies. The cell with an MGS of 8.9 nm gave an elastic modulus range of 95.7–145.9 and 92.5–125.3 GPa using EAM and MEAM MD simulations, respectively. These ranges are in good agreement with the reported experimental range of 108–116 GPa,^52^ where the samples had considerably larger MGS of 54 nm.

**Table 1:** Mean Grain Size (MGS) and Young’s Moduli of Polycrystalline Pure Cu Obtained from Previous Theoretical and Experimental Studies at Various Strain Rates and Temperatures (T)^a^

method	reference	MGS (nm)	*T* (K)	strain rate (s^–1^)	Young’s modulus (GPa)
EAM	Chen^53^	4.65–12.41	300	5 × 10^8^	54–92
EAM	Zhou^54^	2.6–53.1	300	10^8^	25–75*
EAM	Rida^55^	9–24	300	10^8^	55–83
EAM	Xiang^56^	2.9–12.6	300	6.7 × 10^7^	60–112
EMT	Schiotz^57^	3.28–6.56	0	5 × 10^8^	90–120
experimental	Sanders^52^	54	293	10^–4^	108–116
experimental	Cheng^58^	54	297.3	10^–4^	–
experimental	Guduru^59^	23–74	293	4 × 10^–4^	–
EAM	(our results)	4.1	300	10^8^	37.2–67.3
EAM	(our results)	6.1	300	10^8^	79.9–104.7
EAM	(our results)	8.9	300	10^8^	95.7–145.9
EAM	(our results)	10.2	300	10^8^	88.8–104
EAM	(our results)	12.2	300	10^8^	93.3–110.5
EAM	(our results)	16.3	300	10^8^	97.7–136
MEAM	(our results)	8.9	300	10^8^	92.5–125.3

a EMT indicates the many-body effective medium potential approach. For our results, both MEAM and EAM MD simulations are included. Values with an asterisk (*) were obtained using cells with dimensions of one unit cell (one translation, planar cells) along the *z*-axis.

The equilibration of the polycrystalline structures constructed via Voronoi tessellation is crucial for obtaining multigrain simulation cells with structural and mechanical properties comparable to those observed experimentally.^60^ Initially, the total energy of all polycrystalline structures was minimized using the conjugate gradient algorithm with a 10^–10^ eV/Å tolerance for the net atomic forces. To further relax the grain structures, the polycrystals were thermally annealed up to 750 K at a heating rate of 1.5 K/ps, followed by cooling to 300 K at a cooling rate of 1.5 K/ps using a time step of 1 fs at a constant pressure of 1 bar using the isothermal–isobaric *NPT* ensemble. The *NPT* equations of motion were integrated using a leapfrog Verlet algorithm.^61^ The selected pressure was in line with experimental conditions for Cu–Ti alloys.^62^ Previous MD studies in polycrystalline metals, including Cu, have demonstrated that thermally annealing for 250 ps at 0.3–0.5 of the melting temperature of the metal promotes structural relaxation of the grains without allowing excessive grain growth.^63^ To regulate the temperature, a Nosé–Hoover thermostat^64,65^ was used with a time constant of 1 ps, in line with previous MD studies in polycrystalline Cu.^66^ In the case of the bicrystalline Cu undergoing uniaxial tension, following energy minimization, the system is annealed to 300 K at a heating rate of 1.5 K/ps.

Experimentally, Cu samples containing various concentrations of Ti are prepared in powder form after being mixed in plastic canisters with alumina balls for 3 h. Following the mixture process, the powders are sintered at a temperature of 923 K, with a punch load of 50 MPa, a dwelling time of 5 min, and a heating rate of 323 K/min. This process results in Cu–Ti samples with a uniform distribution of Ti.^9^ Accordingly, Ti was introduced randomly into polycrystalline simulation cells prior to equilibration. After equilibration, Ti atoms are expected to occupy their most energetically favorable sites. As an example, in the case where 1.5 at. % Ti was randomly introduced into the polycrystalline Cu cell prior to equilibration, 1.4% of the GB atoms (approximately 2000 atoms) were Ti atoms. After equilibration, 1.6% of the GB atoms were Ti atoms. The 0.2% increase in the number of Ti atoms in the GBs was due to the migration of atoms near the GBs as the system was annealed. During annealing, no Ti migration was observed from the bulk to the GBs. The latter indicates that the temperature reached during equilibration was not sufficient for Ti to overcome the diffusion barriers in the bulk. The corresponding effective Ti concentrations per grain boundary volume for all simulation cells can be found in Appendix A.

For the bicrystalline and polycrystalline Cu–Ti simulation cells, the uniaxial tensile loading along the *y*-axis was conducted at a strain rate of 10^8^ s^–1^, in line with previous MD simulations of Cu.^67^ During the simulations, the cells were kept at a constant temperature of 300 K, and the boundaries in the *x*- and *z*-directions were allowed to adjust to maintain zero pressure. However, 10^8^ s^–1^ is a high strain rate compared with those used in most experimental studies. Thus, it may be necessary to extrapolate the stress–strain curves in the simulations to those obtained at much lower strain rates in the experimental systems.^68,69^

### Energetic Parameters

An important component of these studies is a comparison of the results obtained from MEAM MD simulations and DFT calculations. To distinguish between MEAM and DFT energies, E and  will indicate the MEAM potential energy and the ground state energy minimized by DFT, respectively. To understand the main properties of Ti in Cu GBs, three energetic parameters need to be computed: the (i) segregation, (ii) strengthening, and (iii) separation energies. The segregation energies provide information whether Ti would prefer to be located at the GBs or in the bulk. To calculate the segregation energies of Ti in Cu GB, five substitutional sites are tested, as shown in Figure 1a(i). Previous first-principles theoretical studies demonstrated that substitutional segregation sites are the most favorable for metallic, whereas interstitial sites are the most favorable for nonmetallic impurities.^15,29^ As a result, in this study, only substitutional sites were considered for Ti. The DFT segregation energies  of the substitutional Ti sites in Cu GBs were computed using
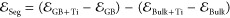
1where  and  are the DFT energies of the GBs and bulk Cu cells, respectively, each containing one substitutional Ti atom.  and  are the respective DFT pristine Cu grain boundary and bulk energies. Negative energies correspond to favorable segregation of Ti at the GB, whereas positive energies correspond to the mixing of Ti in the bulk.

Predictions of the impact of impurities on the grain boundary strength can be made by using the strengthening energy, which provides information whether Ti prefers to be located at the GBs rather than on the sample surface. The DFT strengthening energies , based on the Rice-Wang^70^ model, were computed using
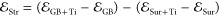
2where  and  represent the DFT energies of the pure Cu (100) surface and Cu surface simulation cells with one Ti atom, respectively. To obtain the Cu surface simulation cells, a slab approach was employed. Starting from the conventional FCC Cu unit cell, a 3 × 3 × 6 translation was performed to create a cell with an extended *z*-dimension. Atoms along the *z*-direction were removed to generate the slab model with the desired slab and vacuum thickness. We set the thickness to be 10.86 Å. A negative value of the strengthening energy means that the impurity will enhance the grain boundary strength, while a positive value implies that the grain boundary will be weakened by the addition of a Ti atom. Similar expressions to those in eqs 1 and 2 were used to compute the MEAM-derived segregation (*E*_Seg_) and strengthening (*E*_Str_) energies, respectively.

Finally, the separation energy indicates whether Ti in Cu GBs promotes or deters fracture initiation. DFT tensile tests use two stretching methods.^71,72^ In this work, we selected a precrack fracture plane based on previous DFT simulations in Σ5 Cu GBs.^29,44^ The separation energies were calculated by subtracting the total energy of the GB cell at a given spacing between the grains from the energy at the equilibrium separation.

## Results

Before MD simulations of uniaxial tensile strain in bicrystalline and polycrystalline Cu were performed, it was important to compare the results of DFT calculations of GB energetics to those obtained from the MEAM interatomic potential. In this section, we compare the MEAM and DFT energies for one Ti atom introduced into the Cu GBs. In addition, we applied uniaxial tension to small Cu–Ti systems and employed DFT to calculate the separation energy for Ti in the Cu GBs.

### Calculations of Segregation and Separation Energies Using DFT and MEAM

In Figure 1a(i), we show the 296-atom (210)[100] Σ5 GB simulation cell for five labeled Ti substitutional sites. For the DFT calculations [see Figure 1a(ii)], segregation sites 1 and 3 had the most favorable (lowest) segregation energies, around −0.35 eV. Previous DFT studies on Al Σ5 GB solutes showed similar negative segregation energies for Ti, around −0.2 eV.^73^ In agreement with DFT, MEAM identified 1 and 3 as the two most favorable Ti substitution sites. For all substitution sites we considered, the MEAM interatomic potential gave segregation energies within 0.15 eV of those obtained using DFT, which, based on previous comparisons between EAM and DFT calculations,^73,74^ we consider reasonable agreement between the two methods.

In Figure 1a(ii), we also display the strengthening energies of GBs in the presence of Ti. We employed a formalism that is frequently used in the literature^44^ to determine the strengthening effect of nonmetallic impurities in Cu GBs. We found that MEAM and DFT give negative strengthening energies for Ti in all substitution sites. Thus, both methods predicted that Ti would have a strengthening effect when introduced into Cu GBs. The two methods were also in good agreement when a different Σ5 twin boundary simulation cell was used [i.e., Σ5 (310)[001] in a simulation cell with 232 atoms, as shown in Figure 1b(i)]. In both Σ5 simulation cells, the substitutional site closest to the center of symmetry of the GBs showed the lowest segregation energy [see Figure 1b(ii)], indicating that our results are relevant to other Σ5 symmetries.

**Figure 2:**
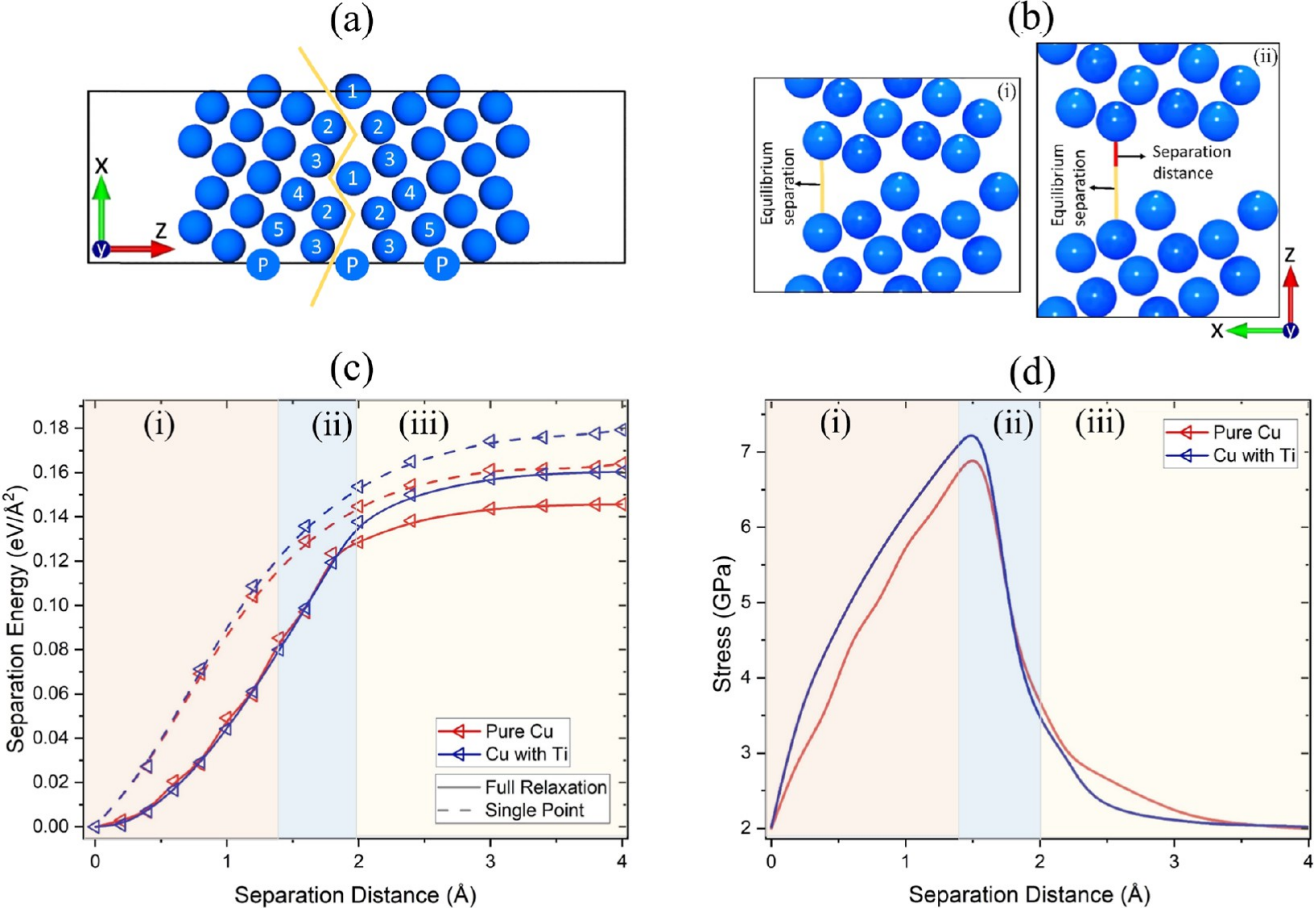
(a) Cu 76-atom (210)[100] Σ5 GB simulation cell used for the DFT calculations of applied unaxial tension. The yellow line shows the fracture plane. (b) Schematic illustrating the Cu GB (i) at the equilibrium separation and (ii) a separation of 2 Å. The yellow and red lines represent the equilibrium and separation distances, respectively. (c) Single point (rigid; no relaxation) and full relaxation DFT calculations of the separation energies, illustrating the energy difference as the separation distance of the GBs is increased for pure Cu and Cu with one substitutional Ti. (d) Hydrostatic stress computed from DFT full-relaxation calculations. The labels (i–iii) denote the three distinctive regions during the DFT uniaxial tension calculations.

In Figure 2a, we show the GB simulation cell used for the DFT uniaxial tension tests. The yellow line illustrates the selected fracture plane. Due to the computational cost of the DFT-based uniaxial tension calculations, we used a smaller cell with 76 atoms. These smaller systems have segregation and separation energies similar to those in Figure 1a(i) (see Appendix B). The effect of Ti on the GB separation energy can be seen in Figure 2c. The separation distance refers to the displacement along the *z*-axis from the equilibrium position, as shown in Figure 2b. We show the separation energies obtained from both single-point (rigid, no atom relaxation allowed) and full-relaxation DFT calculations. The results followed the universal binding energy relation,^75^ where the separation energy increases rapidly for small separations and, at larger distances, reaches a plateau. The smallest separations correspond to the prefracture regime^29^ [region (i) in Figure 2c,d], while, beyond ∼1 Å, the system enters the plastic region [region (ii)], where an intergranular fracture initiates. We define a fracture as the point at which uninterrupted areas of zero electron density are formed within the GB. The calculated separation energy in the case of Cu with a single Ti atom in a periodic cell was approximately 0.02 eV/Å^2^ higher than that for pure Cu. Another interesting point is that for Cu with a single Ti atom, the separation of the GB into two free surfaces (intergranular fracture) occurred at a separation distance of 2.0 Å. On the other hand, in pure Cu, the fracture initiated at a separation distance of 1.8 Å. Finally, in the third deformation stage [region (iii)], the separation energy slowly increased until it plateaued as the remaining long–range interaction forces between the two fracture surfaces tended to zero. The relaxed separation energy curves in the third stage followed the universal binding energy relation.

The tensile strength of the Cu cells with and without Ti can be taken as the maximum tensile stress, as shown in Figure 2d. The fully relaxed DFT calculations show that the tensile strength of the grain boundary with one substitutional Ti was higher compared to the pure Cu grain boundary. According to these findings, doped Cu showed higher mechanical strength compared to that of pure Cu. The observed effect of Ti was in good agreement with previous DFT studies on dopants in Cu, where it was reported that the introduction of transition metals, such as Nb, Mo, and Zr, into Cu can significantly increase the energy for the initiation of intergranular fracture.^44^ In addition, our results were in good agreement with previous theoretical studies in GBs of Au and Fe, which showed that 3d block transition metals can have a beneficial effect on grain boundary cohesion.^18,76^

**Figure 3:**
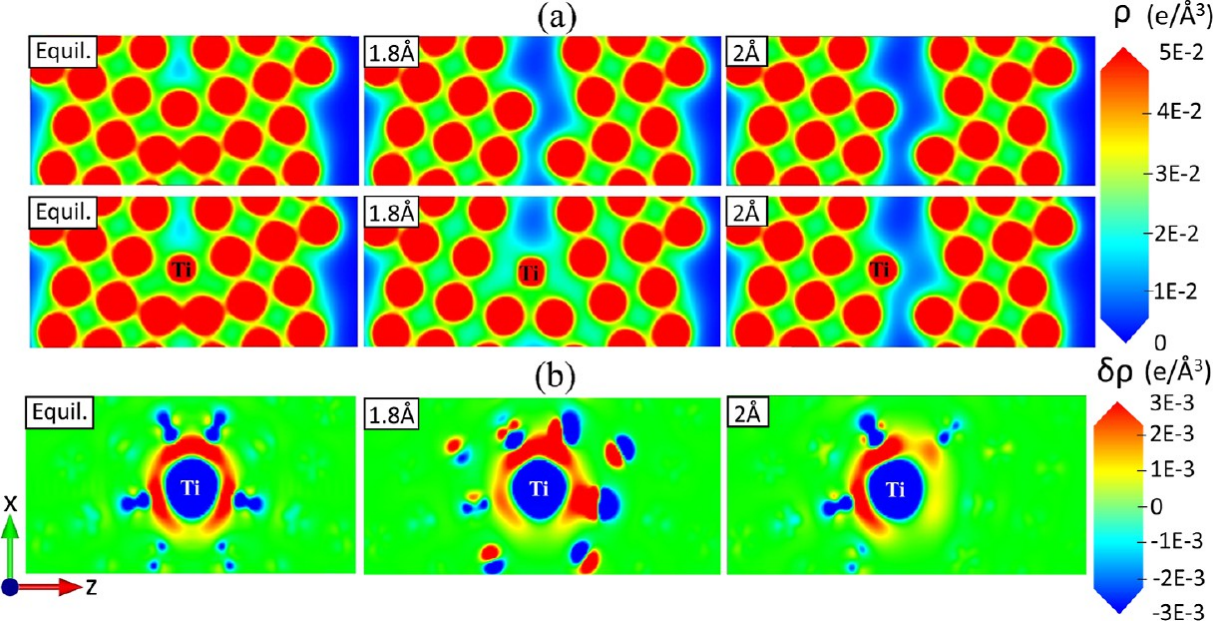
(a) Total electron charge density (ρ) distribution for a pure Cu GB (top) and a Cu GB with a single Ti atom (bottom) at the equilibrium separation (Equil., first column) and separations of 1.8 Å (second column) and 2 Å (third column). (b) 2D contour plots of the differential electron charge density (δρ) for a Cu GB with a single Ti atom at the equilibrium separation (Equil., first column) and at separations of 1.8 Å (second column) and 2 Å (third column). Red (blue) shading corresponds to areas of electron accumulation (depletion). We employed VESTA to visualize the electron charge density distributions.^78^

What is the underlying physical mechanism that gives rise to the strengthening of the Cu GBs with the addition of Ti? In Figure 3a, we show the total charge density distributions (ρ) for a pure Cu GB and a Cu GB with one substitutional Ti atom at site 1 (cf. Figure 2a). The three columns indicate the equilibrium GB separation (first column) and separations of 1.8 Å (second column) and 2 Å (third column). The three columns also represent the three stages of grain boundary decohesion. At a separation of 1.8 Å, a fracture was initiated in the case of the GB cell without Ti, whereas no fracture was observed in the presence of Ti. This effect was attributed to the elongation of the Cu–Cu distance close to the Ti atom. This behavior agrees with the lower separation energy in the case of a Cu GB with Ti, as shown in Figure 2c.

In Figure 3a, we plot the total electron charge density (ρ) distribution contours for a pure Cu GB (top) and a Cu GB with a single Ti atom (bottom). In Figure 3b, we show the differential charge density (δρ) contour maps at the equilibrium separation (first column) and at separations of 1.8 Å (second column) and 2 Å (third column). Ti, which has two extra valence electrons compared to Cu, significantly redistributed the local charge density at a separation of 1.8 Å. At this separation, fracture was also initiated in pure Cu, but not in Cu with Ti (see Figure 3a). From the differential charge density distribution contour plots, we found that the addition of Ti enhanced the charge density near the GB. As noted in previous studies,^73,77^ this charge localization suggests covalent bonding between Ti and the adjacent Cu atoms. Similar covalent bond formation was reported in theoretical studies of Cu–Ti intermetallic compounds, which was attributed to the higher polarizability of Ti.^77^

In accordance with previous DFT studies of segregants in Cu GBs,^29^ electron redistribution with strong relaxation reduces the GB strength. However, in cases where relaxation is minimal, such as Cu GBs with Ti substitutions, charge redistribution increases the local strength. Furthermore, in agreement with previous studies,^29^ dopants in Cu GB that lose electrons are expected to increase the GB strength. Therefore, based on the DFT calculations of Ti in Cu GBs, more energy compared to pure Cu is required to initiate a fracture. Ti enhanced the electron density between the two Cu grains, which led to an increase in the binding energy.

### MD Simulations Using the MEAM Interatomic Potential

#### Bicrystalline Cu–Ti Under Uniaxial Tensile Strain

The results presented in the previous section demonstrated reasonable agreement for the segregation and separation energies of Cu GBs with Ti between the MEAM and DFT methods. We carried out MD simulations using the MEAM interatomic potential in larger and more complex Cu GB structures. We first performed MD simulations of bicrystalline Cu cells (similar to those used in the DFT calculations) undergoing uniaxial tension. The DFT tensile tests revealed that Ti significantly strengthens GBs only for separations ≥1.8 Å. However, these DFT calculations captured only the normal component of the stress and not the shear component, which can induce the formation of topological defects such as dislocations. By characterizing larger bicrystalline GB cells with MD simulations of uniaxial tensile strain, we can investigate the effects of shear stress and dynamics on the yield strength of Cu GBs with varying Ti concentrations.

**Figure 4:**
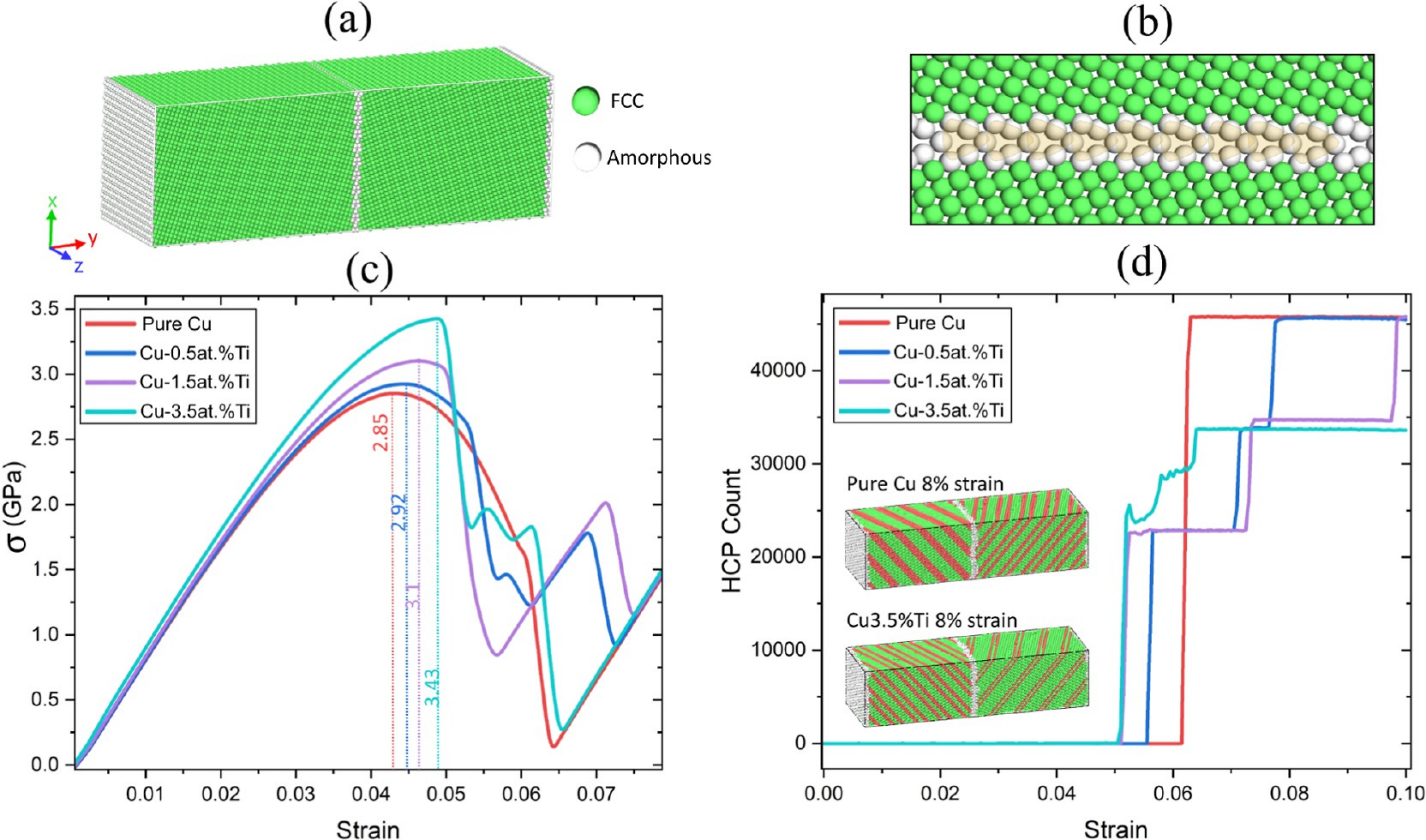
(a) Illustration of the simulation cell used for the MD simulations (using the MEAM interatomic potential) of the bicrystalline (210)[100] Σ5 Cu GB (containing 120,000 atoms) undergoing tensile strain. Strain was applied along the *y*-axis. Atoms shaded green and white indicate the FCC and amorphous regions of the sample, respectively. (b) GB region of the simulation cell. Yellow shading illustrates the Σ5 kite-shaped structural units. (c) Stress versus strain plots obtained from the MD simulations as a function of Ti concentration. The vertical lines indicate the yield strength (in GPa) for each concentration. σ corresponds to the *yy* component of the stress tensor. (d) Number of atoms with HCP local order plotted as a function of strain for several Ti concentrations. The insets provide snapshots of the system at 8% strain for pure Cu (top) and Cu with 3.5 at. % Ti. The red shading indicates atoms with local HCP order. Common neighbor analysis^79^ was used to classify the local crystalline structure surrounding each atom. Structures are visualized using OVITO.^80^

Figure 4a shows the 120,000-atom simulation cell of a bicrystalline Cu GB with the same symmetry as that used in the DFT calculations of Cu GBs undergoing uniaxial tension (see Figure 4b). Constant strain-rate MD MEAM simulations were carried out for pure Cu as well as Cu with 0.5, 1.5, and 3.5 at. % concentrations of randomly distributed substitutional Ti atoms. Uniaxial tension was applied at a constant strain rate after the cell had been equilibrated at a temperature of 300 K. Figure 4c depicts the resulting stress–strain curves, which show that the presence of Ti improved the mechanical properties of the bicrystal, as observed in DFT calculations (see Figure 2). Both the yield strength (vertical lines in Figure 4c) and Young’s modulus increased as the Ti concentration increased.

To better understand the underlying mechanism for the strengthening of Cu GBs, we analyzed the local structural order of the bicrystalline GBs as a function of strain and Ti concentration. As the system was strained beyond yield stress, dislocations were emitted by the GB to release large local concentrations of stress. These dislocations correspond to the formation of stacking faults and twins, which can be identified as planes of atoms with local HCP order. As shown in Figure 4d, the percentage of HCP atoms decreased with increasing Ti concentrations. This result is further illustrated in the insets, where pure Cu possessed a higher density of stacking faults (red-shaded atoms possess HCP order) compared to that for Cu-3.5 at. % Ti at 8% strain. Furthermore, Ti was found to hinder the nucleation of the HCP planes in the case of Cu-3.5 at. % Ti, which resulted in the gradual increase of the number of atoms with HCP symmetry between 5 and 6% strain (see Figure 4d).

#### Polycrystalline Cu–Ti Undergoing Uniaxial Tensile Strain

**Figure 5:**
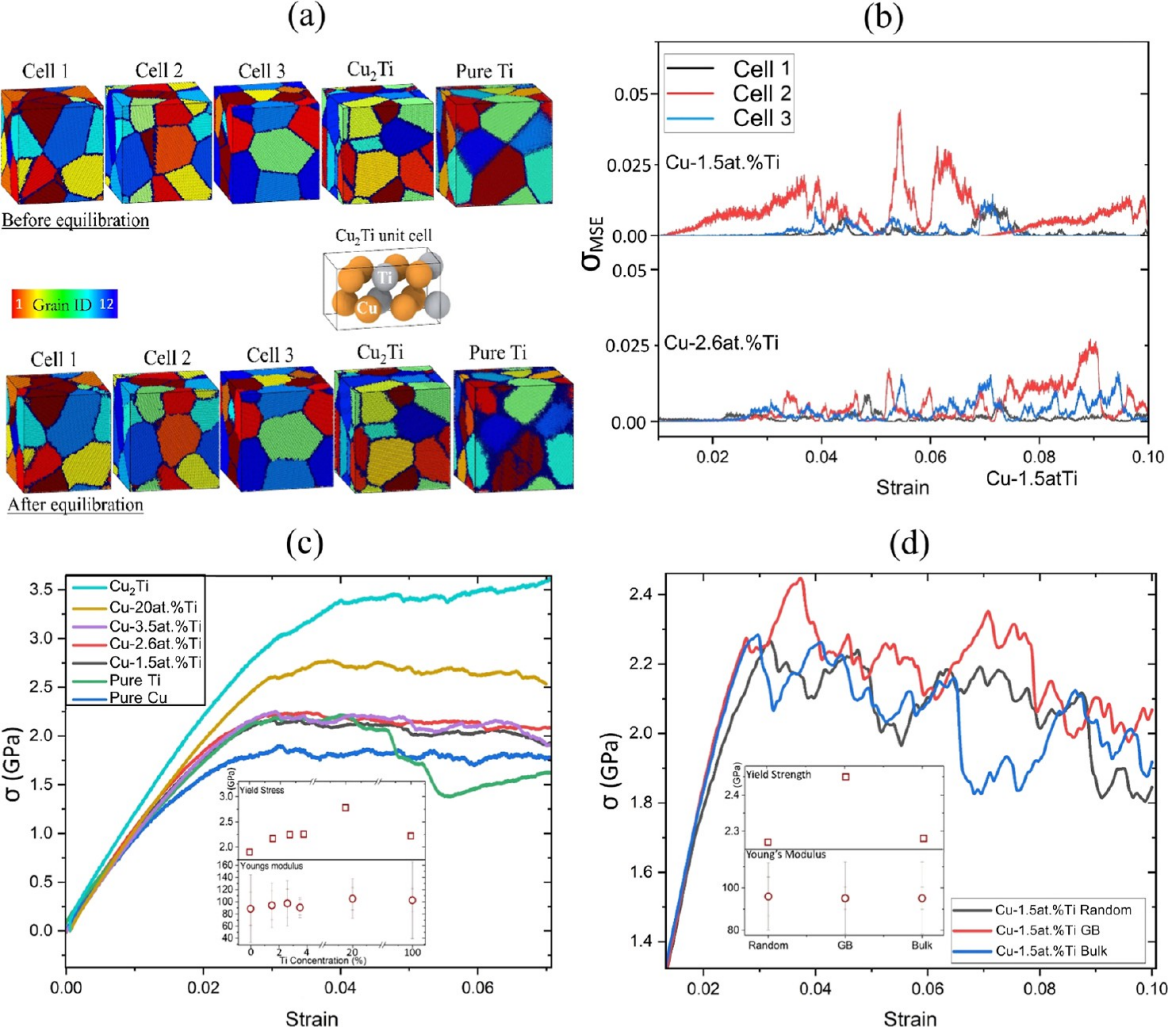
(a) 12-Grain polycrystalline cells for Cu–Ti (cells 1–3), Cu_2_Ti, and pure HCP Ti before (top row) and after (bottom row) equilibration at a temperature of 300 K. The unit cell of bulk Cu_2_Ti is also shown, where brown and gray shading indicate the Cu and Ti atoms, respectively. (b) MSE of the stress from the average value for multiple MD MEAM simulations of uniaxial tension with different random initial distributions of Ti in Cu-1.5 at. % Ti and Cu-2.6 at. % Ti using cells 1–3. (c) Stress plotted versus strain for polycrystalline pure Cu, Cu-1.5 at. % Ti, Cu-2.6 at. % Ti, Cu-3.5 at. % Ti, Cu-20 at. % Ti, and Cu-33 at. % Ti (i.e., the Cu_2_Ti phase). The inset shows the yield strength (top) and Young’s modulus (bottom) for several Ti concentrations. (d) Stress versus strain for Cu-1.5 at. % Ti and different initial distributions of Ti: random, only in the GBs, and only in the bulk. The inset shows the yield strength (top) and Young’s modulus (bottom) for different initial distributions of Ti. σ corresponds to the *yy* component of the stress tensor.

MD simulations of bicrystalline Cu undergoing uniaxial tensile strain showed that Ti hinders the formation of HCP stacking faults, leading to a significant increase in the yield strength. We examined whether similar strengthening occurred for larger and more realistic polycrystalline models of Cu–Ti. We randomly introduced Ti into the polycrystalline simulation cells with different grain distributions. After the addition of the Ti atoms, the polycrystals were equilibrated, as described in the Methods section. In Figure 5a, we display the polycrystalline Cu–Ti cells prior to (top) and after equilibration (bottom). Cells 1–3 correspond to the cells used for the Cu–Ti polycrystals, with Ti concentrations ranging from 1.5 to 20 at. %. Figure 5a also shows the simulation cells for Cu_2_Ti and pure HCP Ti.

For each cell and Ti concentration, three MD simulations of the uniaxial tension were performed. Different initial random Ti distributions were considered for each of the three runs. In Figure 5b, we show the mean-squared error of the stress (MSE; see Appendix A) relative to the average stress over all runs for each of the three simulation cells (cells 1–3 in Figure 5a) for Cu-1.5 at. % Ti and Cu-2.6 at. % Ti. At both concentrations and for cells 1 and 3, the MSE stress was less than 0.02. The MSE in stress continued to decrease with increasing Ti concentration. Interestingly, cell 2 showed the highest MSE in stress for both Ti concentrations. This result can be attributed to the higher fraction of GBs in this cell compared to cells 1 and 3. Even for cell 2, the MSE in stress is <0.05 for Cu-1.5 at. % Ti and <0.025 for Cu-2.6 at. % Ti. Hence, the stress is self-averaged for different initial random distributions of Ti.

In Figure 5c, we show the stress versus strain averaged over multiple runs for pure Cu and Cu-1.5 at. % Ti, Cu-2.6 at. % Ti, Cu-3.5 at. % Ti, and Cu-20 at. % Ti. The 20% concentration was selected as it corresponds to the point in the Cu–Ti phase diagram where FCC Cu transitions to β-Cu_4_Ti.^81^ The stress–strain curve for Cu-33 at. % Ti (Cu_2_Ti phase), using the cell in Figure 5a, is also included as a reference. The inset displays the yield strength and Young’s modulus for pure Cu and Cu with several Ti concentrations. In all cases, the inclusion of Ti increased the Young’s modulus. Furthermore, the yield strength increased with increasing Ti concentration in accordance with the DFT calculations and MEAM MD simulations of bicrystalline Cu–Ti undergoing uniaxial tension. Interestingly, for Ti concentrations higher than 1.5%, the yield strength of Cu–Ti was higher than that of HCP Ti. The mechanical strength of the crystal increased significantly when it transitioned into the Cu_2_Ti phase. This behavior was expected and supported by the solid solution hardening theory for Cu,^82^ which shows that doping Cu with metallic substituents will increase its mechanical strength due to the differing masses of the dopant and host atoms.^29^

We next sought to determine whether the increase in the yield strength of Cu–Ti compared to that of pure Cu can be attributed to the presence of Ti in the GBs. In Figure 5d, we show the stress versus strain for Cu-1.5 at. % when Ti was initially randomly distributed, located only in the bulk, and only in the GBs. In the latter case, Ti was introduced at random sites but only within the grain boundary region, as defined by common neighbor analysis. As shown in the inset of Figure 5d, the inclusion of Ti, either only in the bulk or only in the GBs, did not affect the Young’s modulus for small strains. However, the stress versus strain curve for randomly distributed Ti had a smaller Young’s modulus for strains >2%. Furthermore, when Ti was introduced only in the GB region, we found a considerable increase in the yield strength compared to the case where Ti was distributed randomly or only in bulk (see the inset in Figure 5d). These results illustrate that decorating Cu GBs with Ti atoms would significantly improve the mechanical strength of the crystal.

**Figure 6:**
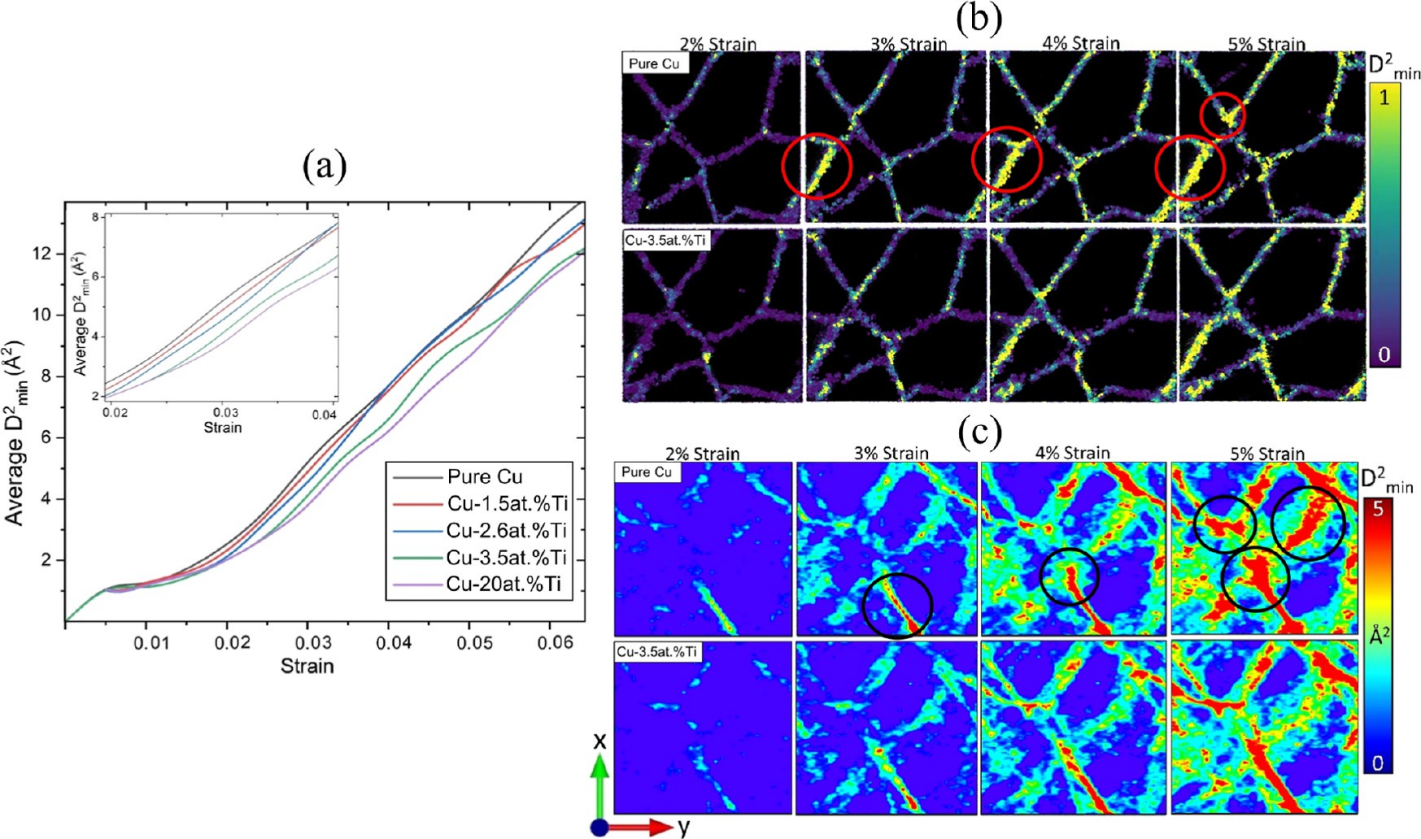
(a) Average nonaffine displacement *D*_min_^2^ plotted versus strain for pure Cu, Cu-1.5 at. % Ti, Cu-2.6 at. % Ti, Cu-3.5 at. % Ti, and Cu-20 at. % Ti undergoing unaxial tension. Only atoms within the GBs are considered. The inset highlights the region from 2 to 4% strain. (b) Spatial distributions of *D*_min_^2^ at a single *z*-slice (*z* = 0) for pure Cu (top) and Cu-3.5 at. % Ti (bottom). (c) *D*_min_^2^ contour plots averaged for all *z* for pure Cu (top) and Cu-3.5 at. % Ti. For pure Cu, triple junctions surrounding smaller grains possess lower values of *D*_min_^2^ [red and black circles in (b,c)].

To better understand the origin of the superior mechanical properties of Cu–Ti, we examined the mechanical response between 2 and 5% strain, approximately where pure Cu and all Cu–Ti alloys reached their yield strength (see Figure 5c). Because polycrystalline cell 1 [Figure 5a(i)] had the lowest MSE in stress among the three cells tested, this cell was used for the following analysis. In Figure 6a, we show the average nonaffine displacement *D*_min_^2^ as a function of strain for pure Cu and Cu with increasing Ti concentration (only atoms within the GBs were included in *D*_min_^2^; see Appendix A for more details). For strains larger than 2%, cells with a higher concentration of Ti had lower *D*_min_^2^ values. The spatial distribution of *D*_min_^2^ for pure Cu and Cu-3.5 at. % Ti at strains between 2 and 5% are shown in Figure 6b,c for a single *z*-slice and averaged over all *z*, respectively. In the case of pure Cu, the GBs of the smaller grains showed higher values of *D*_min_^2^ (see the red and black circles in Figure 6b,c, respectively). The triple junctions and GBs surrounding smaller grains showed considerably smaller *D*_min_^2^ in Cu-3.5 at. % Ti.

**Figure 7:**
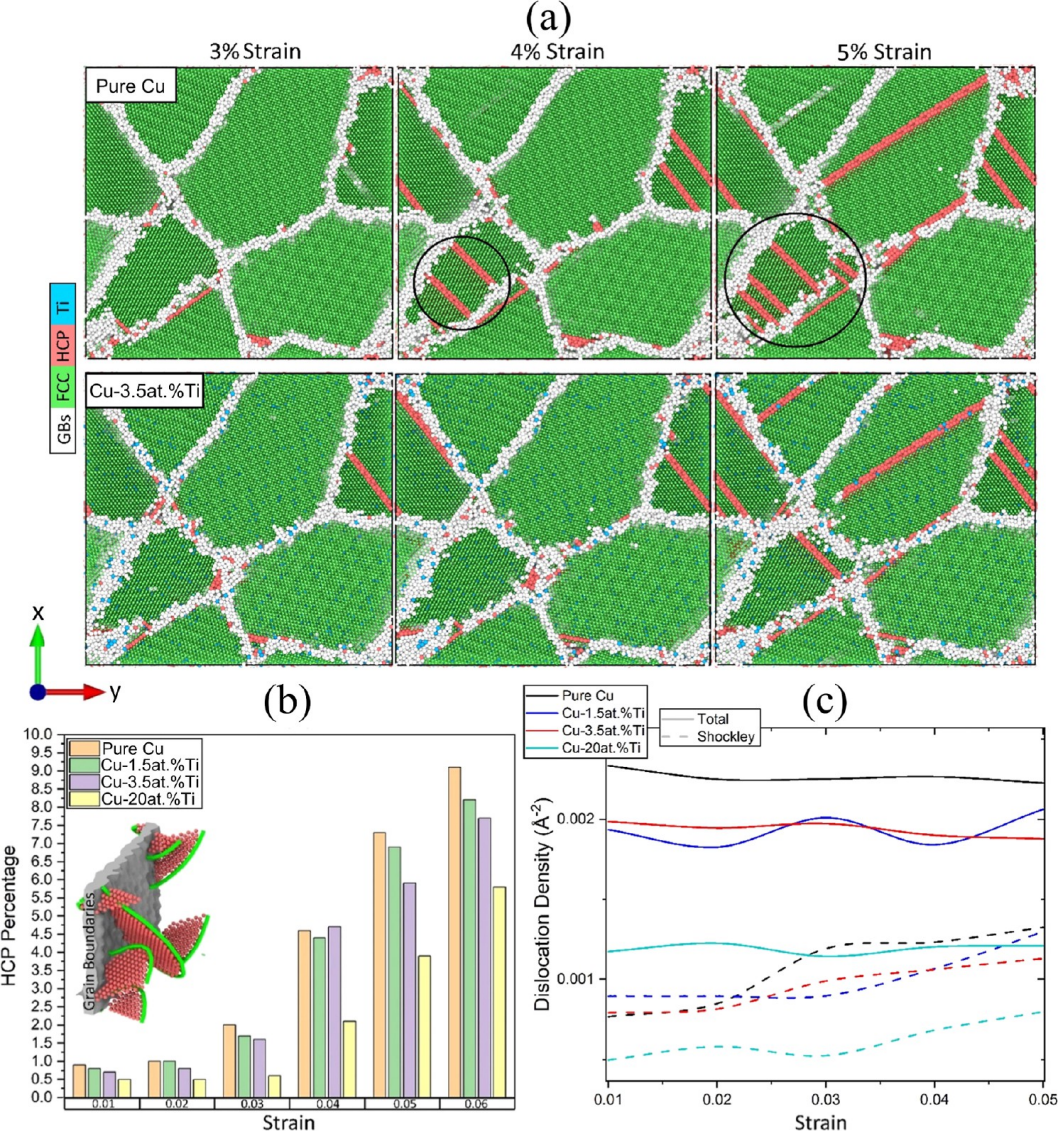
(a) Characterization of the local structural order for (top) pure Cu and (bottom) Cu-3.5 at. % Ti at three strains. Ti (blue) atoms prevent the formation of HCP order (red shading) in smaller grains and reduce the number of grain boundary-induced dislocations (black circles). (b) Percentages of atoms with HCP order for Cu-3.5 at. % Ti at several strains. The inset illustrates the initiation of a stacking fault (HCP atoms in red) via the emission of partial Shockley dislocations (green lines) from the GB (gray region). (c) Total and Shockley dislocation density versus strain for pure Cu, Cu-1.5 at. % Ti, Cu-3.5 at. % Ti, and Cu-20 at. % Ti.

In Figure 7a, we show that intrinsic stacking faults, which are characterized by atoms with local HCP order (red shading), began to nucleate from the GBs and propagate at 3% strain. Consistent with the results from the tensile tests of bicrystalline Cu–Ti, the presence of Ti (blue atoms) prevented the formation of HCP-ordered atoms in smaller grains. Thus, the GBs in Cu-3.5 at. % Ti emitted a lower density of partial dislocations compared to pure Cu (see black circles in Figure 7a). Based on these results, the dislocations emitted by the GBs were likely responsible for both the higher values of *D*_min_^2^ and the lower yield strength of pure Cu compared to the Cu–Ti alloys.

Figure 7b shows the fraction of atoms with local HCP order for Cu–Ti alloys with up to 6% strain. Higher Ti concentrations reduced the number of atoms with HCP order. This effect aligned with what was observed in the MD simulations of bicrystalline Cu–Ti undergoing uniaxial tension. Pure Cu has more HCP atoms at 5% strain than Cu-20 at. % Ti has at 6% strain. The inset in Figure 7b illustrates the initiation of a stacking fault (HCP atoms in red) via the emission of Shockley dislocations by the GBs. We found that Ti inhibited the emission of Shockley dislocations and the nucleation of stacking faults. This dislocation inhibition phenomenon will become more prominent when Ti is introduced in the GBs rather than randomly. This explains the enhanced mechanical properties of Cu–Ti under strain when Ti was introduced only in the GBs (see Figure 5d).

Since dislocations govern the mechanical properties of polycrystals,^73,83^ we examined and quantified the effect of Ti on the dislocation emission process. In Figure 7c, we show the total dislocation density in pure Cu, Cu-1.5 at. % Ti, Cu-3.5 at. % Ti, and Cu-20 at. % Ti as a function of strain. Since partial dislocations (stacking faults) were initially emitted as Shockley dislocations by the GBs,^84^ the Shockley dislocation density is also included for each concentration. Increasing the Ti concentration increased both the total and the Shockley dislocation densities. In pure Cu, the Shockley dislocation density was higher than that for Cu-20 at. % Ti at 3% strain. Thus, Ti is expected to reduce twinning, a process during which a part of the metal undergoes shear deformation along a preferred slip plane. The results in Figure 7a,b are consistent with twinning, where the addition of Ti was found to alter the crystal’s ability to generate HCP planes. A similar finding was reported in bicrystalline Al through MD simulations, where the inclusion of Mg in the GB hindered the nucleation of dislocations, leading to an increased yield strength.^85^

Based on our analysis and taking into account the already established role of dislocation emission in nanocrystalline FCC metals,^83^ we have identified a mechanism where the inclusion of Ti atoms improves the mechanical properties of the samples under tensile loading. Initial plastic deformation occurs under tensile loading through the nucleation of partial dislocations emitted by the GBs at a strain of ∼1.5%. The continual process of partial dislocation nucleation from GBs and annihilation of GBs at opposite ends of the grains causes pronounced local distortion close to the GBs. With the addition of Ti, the distortion is less pronounced. Ti, when introduced into the GBs, increases the energy needed for the GBs to slide and emit partial dislocations. Thus, the inclusion of Ti substantially decreases the density of the emitted partial dislocations by the GBs, leading to an increase in the yield stress. The latter observation is in agreement with the Hall–Petch equation,^86,87^ which predicts that as the stress needed for the activation of the dislocations increases, the yield strength also increases.

Our MD simulations demonstrate a change in the dislocation dynamics and mechanical response of Cu–Ti alloys as the Ti concentration varies. The MD simulations show that with an increase in Ti concentration, there is a corresponding reduction in the formation of stacking faults and, thus, an increase in the stacking fault energy (SFE). The latter result is consistent with previous studies,^88,89^ which emphasize that 3D transition metals like Ni, when introduced into Cu at concentrations up to 5% are expected to increase the strength and hardness of the metal due to solid–solution hardening, leading to an increase in the SFE.

## Summary and Conclusions

Previous studies have mainly used DFT calculations to investigate the role of metallic solutes in the GBs of Cu in determining its mechanical properties. To better understand the effect of Ti on the mechanical properties of Cu, we employed both DFT calculations and MEAM MD simulations, which enabled studies of tensile deformation over a range of length scales. We first compared the results from the MEAM potential for the most energetically favorable Ti substitution sites in Cu GBs with the results from the DFT calculations. For both the DFT calculations and MEAM MD simulations, substitutional Ti preferred to segregate at the GB rather than in the bulk of Cu. DFT calculations of tensile deformation show that Ti at the GBs induced local charge localization, which increased the maximum stress and the separation and strengthening energies of the GB. An interesting future direction would be to examine whether other elements with properties similar to Ti, in terms of the number of valence electrons or atomic radius, when added to Cu GBs would cause similar charge localization and increases in the maximum stress, segregation, and separation energies.

The changes in the mechanical properties of large bicrystalline Cu cells in response to the addition of Ti were studied by using MEAM MD simulations of uniaxial tension. Similar to the results obtained from DFT calculations of uniaxial tension, the addition of Ti increased the yield strength of the crystal due to the inhibition of stacking faults emitted by the GBs. This phenomenon was also present when polycrystalline systems were considered. MEAM MD simulations using nanocrystalline multigrain simulation cells showed that the presence of Ti prevents the emission of partial Shockley dislocations from GBs, which reduces local distortions. This effect became more pronounced as the Ti concentration increased and when all Ti atoms were introduced at the GBs of the polycrystal. Thus, the inclusion of Ti resulted in a significant increase in the yield strength and Young’s modulus of Cu polycrystals compared to those of pure Cu polycrystals. These mechanical properties were further improved at higher Ti concentrations.

Our findings suggest that the addition of Ti to Cu has a significant effect on the yield strength and Young’s modulus of the material. Thus, grain boundary segregation engineering of polycrystalline Cu with Ti solutes can be used to enhance the material’s durability and broaden its industrial applications. However, due to the intrinsic limitations of the time and length scales of MD simulations, the unrealistically high deformation rates used in MD simulations were many orders of magnitude higher than those used in experiments. Alongside the current data, future work should focus on extrapolating the mechanical properties of Cu–Ti to significantly slower strain rates. In addition, further investigations are needed for a broader range of Cu–Ti alloys. Finally, to simulate polycrystalline Cu–Ti cells with mean grain sizes closer to those studied experimentally, reliable and less computationally demanding MEAM potentials for modeling polycrystalline Cu–Ti will be required.

## Supporting Information Available

MEAM formalism, structural analyses, simulation parameters, DFT calculations, and MEAM MD simulations of segregation of Ti in Cu grain boundaries (PDF)
