# Supplementary material for: Modeling the Effects of Varying the Ti Concentration on the Mechanical Properties of Cu–Ti Alloys
Source: ACS Omega. 2024 Feb 19;9(9):10286–98. doi: 10.1021/acsomega.3c07561 (PMC10918840; doi:10.1021/acsomega.3c07561)
Supplement: Supplementary file 1 — ao3c07561_si_001.pdf [file ao3c07561_si_001.pdf]

Modeling the Effects of Varying Ti Concentration on the Mechanical Properties of Cu-Ti Alloys

Vasileios Fotopoulos,[*,†] Corey S. O'Hern,[‡,¶,§] Mark D. Shattuck,[‖] and Alexander L. Shluger[†,⊥]

†*Department of Physics and Astronomy, University College London, Gower Street, London WC1E 6BT, U.K.*

‡*Department of Mechanical Engineering & Materials Science, Yale University, New Haven, Connecticut 06520, United States*

¶*Department of Physics, Yale University, New Haven, Connecticut 06520, United States*

§*Department of Applied Physics, Yale University, New Haven, Connecticut 06520, United States*

‖*Benjamin Levich Institute and Physics Department, The City College of the City University of New York, New York 10031, United States*

⊥*WPI-Advanced Institute for Materials Research (WPI-AIMR), Tohoku University, Sendai 980-8577, Japan*

E-mail: vasileios.fotis.19@ucl.ac.uk

Appendix A: Computational Methods

MEAM Formalism

This section describes the MEAM interatomic potential that was employed in the molecular dynamics simulations of Cu-Ti alloys. To model the Cu-Cu, Cu-Ti, and Ti-Ti interactions, we used a recently developed MEAM potential to study Cu, Ti, and N interfaces.[1] The mathematical foundations for MEAM interatomic potentials were described by Baskes, et al.[2] In the MEAM formalism, the total energy of the system is

$$\mathrm{E} = \sum_{i} F_i(\rho_i) + \frac{1}{2}\sum_{i}\sum_{i \neq j} S_{ij}\phi_{ij}(r_{ij}), \tag{S1}$$

where $i, j = 1, \ldots, N$ label the atoms, r_{ij} is the distance between the atoms i and j, and ϕ_{ij} is their pair potential. In general, the screening factor represents the influence of nearest neighbor atoms k on the pair interactions between atoms i and j. S_{ij} is the screening factor for second nearest neighbor (2NN) interactions, and the screening factor for (1NN) interactions is unity. The screening from the kth atom for the interaction between atoms i and j is determined using an ellipse in the x-y plane with the long axis of the ellipse (i.e. the x-axis) aligned with the separation vector between atoms i and j and the three atoms i, j, and k lie on the boundary of the ellipse. The ellipse equation is given as:

$$x^2 + C^{-1}y^2 = \frac{r_{ij}^2}{4}, \tag{S2}$$

where x and y are the coordinates of the kth atom. As described by Lee, et al.,[3] the upper and lower limits of the screening function can be defined through the minimum and maximum limit parameters $C_{(\min)}$ and $C_{(\max)}$. If an atom k is located outside of the ellipse defined by $C_{(\max)}$, it does not affect the pair interaction between atoms i and j atoms and the screening factor is unity. If $C < C_{(\min)}$, the kth atom fully screens the pair interaction, and the screening factor $S_{ij} = 0$. The screening factor varies smoothly over the range

$C_{(\max)} > C > C_{(\min)}$. The corresponding many-body screening factor S_{ij} between the i and j atoms can then be expressed as the product of the screening factors resulting from all neighboring atoms k. In the case of pure Cu or pure Ti, two types of limit parameters need to be defined: $C_{(\min)}$ and $C_{(\max)}$ for both Cu and Ti. In the case of binary systems, i.e. Cu-Ti alloys, eight limit parameters are needed, as shown in Table S1.

Embedding function

$$F(\rho_i) = \mathrm{AE}_C \left(\frac{\rho_i}{\rho_e}\right) \ln\left(\frac{\rho_i}{\rho_e}\right), \tag{S3}$$

where E_C represents the cohesive energy, A is an adjustable parameter, and ρ_e is the background electron density of the reference crystal structure. ρ_i is the background electron density, which is composed of the partial electron densities $\rho_i^{(0)}$, $\rho_i^{(1)}$, $\rho_i^{(2)}$, $\rho_i^{(3)}$ that represent the contributions from the s, p, d, and f electron densities, respectively. Each partial electron density is computed as follows:

$$\rho_i^{(h)} = \rho_{sc} \exp\left[\beta^{(h)}\left(\frac{R}{R_e - 1}\right)\right], \tag{S4}$$

where $\beta^{(h)}$ (with $h = 0, 1, 2, 3$ for the s, p, d, and f electron shells) are the decay lengths that control the strength of the electronic interactions. ρ_{sc} is the scaling factor of the electron density, R gives the distance to the 1NN atoms, and R_e is the equilibrium 1NN distance of atoms in the reference structure. The total electron density at site i is then written as:

$$\rho_i = \rho_i^{(0)} \Gamma(P_i), \tag{S5}$$

where

$$P_i = \sum_{h=1}^{3} t_i^{(h)} \left[\frac{\rho_i^{(h)}}{\rho_i^{(0)}}\right]^2, \tag{S6}$$

$$\Gamma(P_i) = \frac{2}{1+e^{-P_i}}, \tag{S7}$$

and $t_i^{(h)}$ are the weighting factors for the contributions to the electron density from each electron shell given in Table S1.

Table S1: **MEAM Parameters for Binary Cu-Ti, Pure Cu, and Pure Ti Systems**

	**Cu-Ti** (Binary System)		**Pure Cu**	**Pure Ti**
E_C(eV)	4.40	E_C(eV)	3.54	4.87
R_e(Å)	2.66	R_e(Å)	3.61	2.92
$C_{(\text{min})}$(Cu-Cu-Ti)	0.3	A	0.91	1.19
$C_{(\text{min})}$(Ti-Ti-Cu)	1.03	$\beta^{(0)}$	3.68	1.58
$C_{(\text{min})}$(Cu-Ti-Cu)	0.91	$\beta^{(1)}$	4.30	0.08
$C_{(\text{min})}$(Cu-Ti-Ti)	1.12	$\beta^{(2)}$	5.75	2.89
$C_{(\text{max})}$(Cu-Cu-Ti)	3.93	$\beta^{(3)}$	0.12	0.002
$C_{(\text{max})}$(Ti-Ti-Cu)	3.31	$C_{(\text{min})}$	0.51	0.89
$C_{(\text{max})}$(Cu-Ti-Cu)	3.72	$C_{(\text{max})}$	1.92	2.85
$C_{(\text{max})}$(Cu-Ti-Ti)	1.69	$t_i^{(1)}$	2.32	5.55
ρ_0(Ti)/ρ_0(Cu)	1.00	$t_i^{(2)}$	6.94	6.79
		$t_i^{(3)}$	6.00	-2.05

Structural Analyses

We performed structural analyses to understand the effect of Ti on the formation of defects such as dislocations in polycrystalline Cu under tensile strain. The dislocation distribution and density were calculated using the dislocation extraction algorithm (DXA).[4,5] DXA allows us to identify all dislocation line defects in crystals, determine their Burgers vectors, and output line representations of the dislocations. The common neighbor analysis (CNA) method[6] was used to classify the local crystalline structure surrounding each atom, i.e., FCC, BCC, HCP, or amorphous. The method compares the coordinates of the first and second nearest neighbor atoms to those of the central atom of interest to determine the local crystalline order.

To quantify and visualize the local deformation of Cu-Ti under uniaxial tensile loading, we calculated the nonaffine displacement field. The nonaffine displacement of a given atom describes the mean-square deviation between the displacements of its first nearest neighbor atoms and the displacements of the same atoms undergoing a given strain tensor ϵ_{ij}:[7]

$$\mathrm{D}^2(t, \Delta t) = \sum_n \sum_i \left(r_n^i(t) - r_0^i(t) \; - \sum_j (\delta_{ij} + \epsilon_{ij}) \times [r_n^j(t-\Delta t) - r_0^j(t-\Delta t)] \right)^2 , \tag{S8}$$

where i and j denote the indices of the atomic coordinates, and the index n runs over the atoms within the interaction cutoff of second nearest neighbor atoms. The ith component of the position of the nth atom at time t is denoted by $r_n^i(t)$. $n = 0$ detonates the reference atom and δ_{ij} is the Kronecker delta. The strain ϵ_{ij} that minimizes the expression in Eq. S8 is computed using a least-squares method as follows:

$$\epsilon_{ij} = \sum_k X_{ik} Y_{jk}^{-1} - \delta_{ij}, \tag{S9}$$

where

$$X_{ij} = \sum_n \left([r_n^i(t) - r_0^i(t)] \times [r_n^j(t-\Delta t) - r_0^j(t-\Delta t)] \right), \tag{S10}$$

and

$$Y_{ij} = \sum_n \left([r_n^i(t-\Delta t) - r_0^i(t-\Delta t)] \times [r_n^j(t-\Delta t) - r_0^j(t-\Delta t)] \right). \tag{S11}$$

By combining Equations S8 and S9, we can calculate the local deviation from an affine deformation ($\mathrm{D}^2_{\mathrm{min}}$) over the time interval Δt.

Once Ti was introduced in the Cu polycrystals, multiple molecular dynamics simulations of uniaxial tension were carried out to determine averaged stress versus strain curves. We calculated the mean-squared error (MSE) in stress as a function of strain as follows:

$$\sigma_{\mathrm{MSE}} = l^{-1} \sum_{i=1}^{l} (\sigma_i - \bar{\sigma}_i)^2, \tag{S12}$$

where l is the number of simulations, σ_i is the stress of the ith simulation, and $\bar{\sigma}_i$ is the average stress over all simulations.

Simulation Parameters

Table S2 summarizes the parameters for the different simulation cells. Table S3 lists the grain boundary sizes and the effective Ti concentration per grain boundary volume.

Table S2: **Number of Atoms and Volume for Each Simulation Cell**

Cell	Atoms	Volume (nm^3)
Bulk Cu	108	1.09×1.09×1.09
(210)[100] Σ5 GB	76	0.81×0.73×2.44
(210)[100] Σ5 GB	152	1.63×0.73×2.44
(021)[100] Σ5 GB	232	1.13×0.73×4.41
(210)[100] Σ5 GB	296	1.63×0.73×5.18
Surface (100)	108	1.09×1.09×2.09
(210)[100] Σ5 GB	120,000	24.31×8.08×8.08
Polycrystal Σ5 GB	665,500	20×20×20

Table S3: **Effective Ti Concentration Per Volume of GB for Each Simulation Cell**

Cell	Atoms	at.%Ti	Ti atoms/GB volume (atoms/nm^3)
(210)[100] Σ5 GB	76	1.32	2.23
(021)[100] Σ5 GB	232	0.43	1.52
(210)[100] Σ5 GB	296	0.34	0.95
(210)[100] Σ5 GB	120,000	1.5	1.57
Polycrystal	665,500	1.5	0.47

Appendix B: DFT Calculations and MEAM MD Simulations of Segregation of Ti in Cu Grain Boundaries

In this appendix, we study the cell size needed to minimize the difference between the segregation and strengthening energies from the DFT calculations and MEAM interatomic potential. In Figure S1, we show several Ti substitutional segregation sites in the Cu $\Sigma5$, 53.1° (210)[100] grain boundary [Figures S1a(i) and b(i)]. Using both DFT calculations and MEAM interatomic potential, segregation site 1 gave the lowest segregation energies [Figures S1a(ii) and b(ii)]. In addition, for both cell sizes, the DFT calculations and MEAM interatomic potential gave negative strengthening energies for all five substitutional sites, indicating that Ti will have a strengthening effect when introduced in this grain boundary.

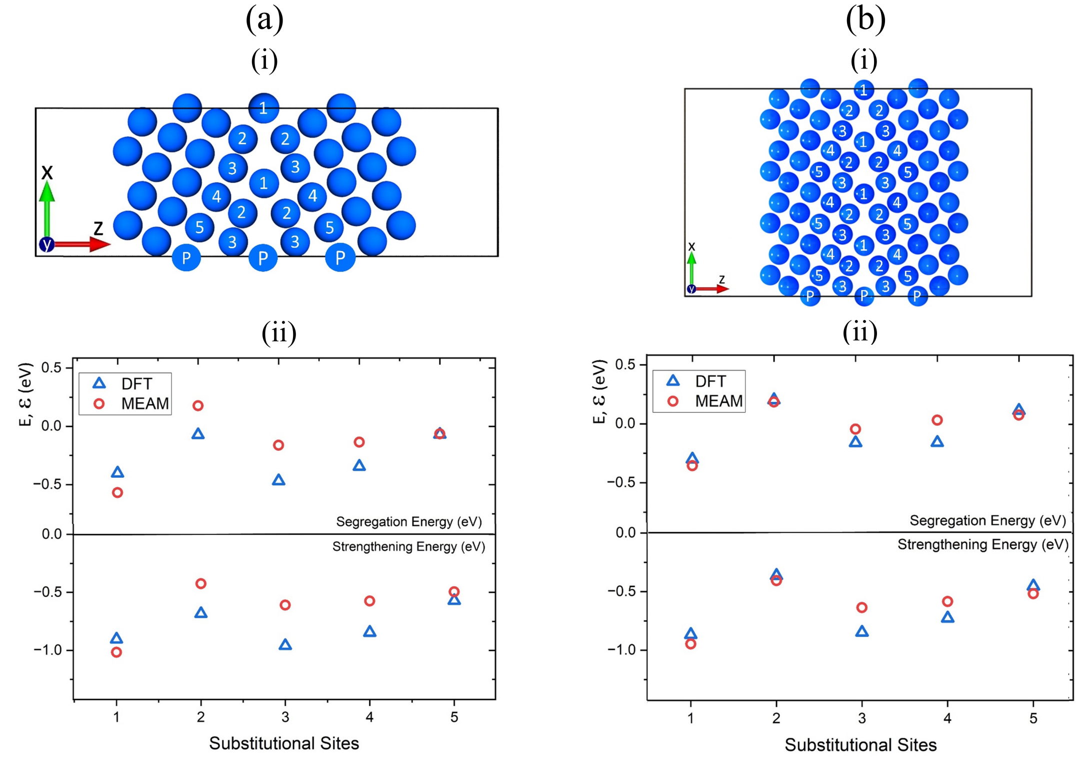

Figure S1: (a) (i) 76-atom (210)[100] Σ5 grain boundary simulation cell. Cu atoms are shown in blue, and the numbered atoms correspond to the substitutional Ti sites. Atoms labeled with 'P' represent atoms that are periodically translated. (ii) Comparison of Ti segregation (top) and strengthening energies (bottom) obtained using DFT calculations and the MEAM interatomic potential for the five substitutional sites. (b) (i) 152-atom (210)[100] grain boundary simulation cell. (ii) Comparison of Ti segregation (top) and strengthening energies (bottom) from DFT calculations and the MEAM interatomic potential.

References

(1) Miraz, A. S. M.; Dhariwal, N.; Meng, W.; Ramachandran, B. R.; Wick, C. D. Development and application of interatomic potentials to study the stability and shear strength of Ti/TiN and Cu/TiN interfaces. *Materials & Design* **2020**, *196*, 109123.

(2) Baskes, M.; Nelson, J.; Wright, A. Semiempirical modified embedded-atom potentials for silicon and germanium. *Physical Review B* **1989**, *40*, 6085.

(3) Lee, B.-J.; Baskes, M.; Kim, H.; Cho, Y. K. Second nearest-neighbor modified embedded atom method potentials for bcc transition metals. *Physical Review B* **2001**, *64*, 184102.

(4) Stukowski, A.; Albe, K. Extracting dislocations and non-dislocation crystal defects from atomistic simulation data. *Modelling and Simulation in Materials Science and Engineering* **2010**, *18*, 085001.

(5) Stukowski, A.; Bulatov, V. V.; Arsenlis, A. Automated identification and indexing of dislocations in crystal interfaces. *Modelling and Simulation in Materials Science and Engineering* **2012**, *20*, 085007.

(6) Faken, D.; Jónsson, H. Systematic analysis of local atomic structure combined with 3D computer graphics. *Computational Materials Science* **1994**, *2*, 279–286.

(7) Falk, M. L.; Langer, J. S. Dynamics of viscoplastic deformation in amorphous solids. *Physical Review E* **1998**, *57*, 7192.
